# Agnostic Tendency in Doctors

**Published:** 1885-01-20

**Authors:** 


### AGNOSTIC TENDENCY IN DOCTORS.

The New York Medical Record seems to regard as probably true, "that Christian reverence and faith are being supplanted, in a measure, by indifference and agnosticism on the part of physicians." This is attributed to the fact that "the doctor sees so many promises fail; hears so much that turns out false; reads so much that proves to be rubbish that, as he passes on in life, he is more and more inclined to base his convictions only on that he has himself seen or known. What wonder that he sometimes doubts the beneficence of Mercy, and the utility of Piety, or questions if the human wrecks that he meets have been ennobled with immortality, or stamped in the image of God"

If this be true it is certainly a matter of deep regret. Shall he who has most to do with human suffering and misery, and who most sees the need and propriety of the blessed compensation for which we strive, and hope and pine, in the next stage of existence; shall we, more than others, doubt the immortality of the soul, or fail to trust in the power and beneficence of that Great Being of whose existence and wisdom we see so much evidence in the physiological anatomy of the human frame? W.

